# AI Ethics, Ethics Washing, and the Need to Politicize Data Ethics

**DOI:** 10.1007/s44206-022-00013-3

**Published:** 2022-08-02

**Authors:** Gijs van Maanen

**Affiliations:** Molengraaff Institute for Private Law, Utrecht University, Utrecht, The Netherlands

**Keywords:** AI ethics, Ethics washing, Political theory, Geuss, Casuistry

## Abstract

Many commercial actors in the tech sector publish ethics guidelines as a means to ‘wash away’ concerns raised about their policies. For some academics, this phenomenon is reason to replace ethics with other tools and methods in an attempt to make sure that the tech sector does not cross any moral Rubicons. Others warn against the tendency to reduce a criticism of ‘ethics washing’ into one of ethics simpliciter. In this essay, I argue firstly that the dominant focus on principles, dilemmas, and theory in conventional ethical theories and practices could be an explanation of it lacking resistance to abuse by dominant actors, and hence its rather disappointing capacity to stop, redirect, or at least slow down big tech’s course. Secondly, drawing from research on casuistry and political philosopher Raymond Geuss, this essay will make a case for a question, rather than theory or principle-based ethical data practice. The emphasis of this approach is placed on the acquisition of a thorough understanding of a social-political phenomenon like tech development. This approach should be replenished with one extra component to the picture of the repoliticized data ethics drawn so far: the importance of ‘exemplars,’ or stories. Precisely the fact that one should acquire an in-depth understanding of the problem in practice will also allow one to look in the past, present, or future for similar and comparable stories from which one can learn.

## Introduction

The growing number of ethical guidelines, ethical committees, and ethicists that can be found in both the public and private sectors nowadays prompts researchers within the computer and data science community to question the role that ‘ethics’ plays within the tech industry (Green, 2021; Jobin et al., 2019; Metcalf et al., 2019; Morley et al., 2019; Schiff et al., 2020). The idea and practice of ethics, according to critics, is used by companies to wash away the concerns raised by a company’s behavior or a techno-political crisis (Wagner, 2018). Ethics, also, allows one to strategically ‘shop’ for the principles that limit one’s action as little as possible while simultaneously presenting oneself as contributing towards the common good. The pessimistic implication of this behavior could be to look for alternative tools and methodologies to make sure that the tech sector does not cross any moral Rubicons. Think for instance about replacing ethics for science and technology studies (STS) (Sloane, 2019), new forms of ‘data justice’ (Taylor, 2019), or about playing safe and falling back to a human rights regime (Yeung et al., 2020). Some of the more optimistically inclined scholars in this debate beg to differ, and warn against the tendency to reduce a criticism of ‘ethics washing’ into one of ethics simpliciter (Bietti, 2020). While these optimists are right in emphasizing the danger of conflating a company’s usage of ethics, and ethics as a valuable and important activity, the apparent incapacity of ‘ethics’ and ethicists to resist against this ‘abuse’ might be indicative for a limitation for relying on ethics as a tool, method, or attitude in this particular context (Maanen, 2020).

In this essay, I argue that the focus on principles, dilemmas, and theory in popular ethical theories and practices could be one of the factors that explain its lacking resistance to abuse by dominant actors. Section 1 introduces the cornerstone of most types of (tech) ethics: ‘the’ ethical question. In Sect. 2, I introduce the problem this essay deals with, with the help of an influential AI ethics example. I suggest here that the discussed work exemplifies a particular way of doing ethics characterized by the formulation of moral principles and frameworks. Section 3 speculates on why there is a seeming abundance of principle-oriented works on data ethics with the help of research done by Albert R. Jonsen and Stephan Toulmin (Jonsen & Toulmin, 1988). The authors show that for the biggest chunk of Western history, a practice-oriented mode of moral reasoning was dominant. This form of ethics, however, lost its popularity in the seventeenth century, to be replaced by a more theory and principle-focused alternative. Ever since, the dominant approach in ethics or moral philosophy is one focusing on theories, principles, and generalizations. This, however, has disadvantages, to be discussed in Sect. 4, where I draw from the work of Raymond Geuss to make a case for a question, rather than theory or principle-based ethical practice. The emphasis of this approach is placed on the acquisition of a thorough understanding of social-political phenomena (like tech development). In two Sects. 5 and 6, I draw out two consequences of the practice-oriented approach put forward so far: the need to do empirical research, and the importance of ‘exemplars’ or stories. Section 7 relates the argument developed so far to similar (academic) attempts to politicize the practice of data science and ethics. The question answered here is to what extent the label of ‘ethics’ still characterizes my proposal.

For both heuristic and methodological reasons, the scope of this essay is limited. It focuses on one type of ethics, which is placed in opposition to practice-oriented alternatives. This kind of ethics is primarily being practiced by academics,^1^ though it is more and more being co-opted by industry and, as such, also practiced there (Green, 2021; Hu, 2021). Empirically speaking, the boundaries between ethics as academic inquiry, and ethics as practiced in industry, are thus hard to draw. Because of this interminglement of academia and industry, I argue near the end of this paper for the explicit acknowledgement of the situatedness of one’s ethical research, including its susceptibility to (mis)use by others. By mainly engaging in ethics in a principled and constructive manner without acknowledging its politics,^2^ one disregards and by doing so *accepts* the mutual dependencies existing between research and commerce. It is lastly good to note that this essay is limited to a genealogy-based explanation for AI ethics’ susceptibility to be washed by the private sector. Other possible explanatory factors—e.g., academic funding structures; lobbying power—are hinted at but not studied extensively for this essay.

## The Ethical Question

Most ethics attempt to answer the ethical question of what one should do, or how one should live.^3^ Ethics is then considered the practice or tool to be used to approach an answer to this question. ‘Within’ ethics, usually three different approaches to formulating an answer to the ethical question can be identified. There are deontological approaches focusing on rules and duties, teleological ones emphasizing the importance of evaluating consequences, and others that focus on virtues (Ananny, 2016, p. 94). Deontological approaches within the context of AI ethics attempt to codify ethical theories and principles, and conceive ethics as intimately connected to policy and regulation. Teleological or consequentialist ones attempt to predict or estimate the risks new technologies could pose and, based on such evaluations, determine if how and in what way a technology should be implemented. Virtue ethicists, lastly, are interested in how practitioners incorporate ethical norms in the design process. Forms of ‘Value Sensitive Design’ (VSD) could be associated with such a take on the ethical question (Greene et al., 2019, pp. 2123–2124; Jacobs et al., 2021).

The differences between the three different approaches are large—theoretically, practically, and popularity-wise. What makes them, in the end, ‘subareas’ of ethics is their explicit or implicit acceptance of the ethical question, and the attempt to supply it with answers. Popularity-wise, the most dominant AI ethics approach in academia appears to be ‘deontology,’ wherein answers to the ethical question are put in the form of lists of principles, values, and maxims (Hagendorff, 2020, p. 112). This makes it a ‘law conception of ethics,’ which either aims to be like law, or to be one of the ingredients to be inserted into law’s construction (Rességuier & Rodrigues, 2020). Throughout this essay, this prioritization of the ethical question, and the most prevalent deontological answers to it, will be questioned.

## The Problem of Ethics (Washing)

The writing and publishing of lists of ethical principles that should help academics and practitioners determine ethical boundaries is big business.^4^ Not only do public and private actors for various reasons try their very best to keep up with this trend (Schiff et al., 2020; Taylor & Dencik, 2020; Yeung et al., 2020), academics also have a weak spot for the regular publishing of lists of important moral principles that should (help to) regulate individual and collective behavior in the tech world. One example of such a contribution is a paper written by Luciano Floridi and Josh Cowls (Floridi & Cowls, 2019). It starts with the diagnosis that there are simply too many ethical lists and guidelines floating around, which are causing confusion, information overload, and even stimulate the kind of ‘ethics shopping’ we previously linked to the phenomenon of ethics washing.^5^ What, then, to do about this problem? Floridi and Cowls read and analyzed six of these guidelines, found a lot of similarities, and put forward a framework encompassing the principles listed, plus one extra they thought to be missing. Doing AI ethics, here, is thus the analysis of principles that relate to a particular technology (AI). Floridi and Cowls argue that their principles ought to serve as the ‘architecture’ used to build policies around the globe, with as small caveat that it be, “(…) broadened to incorporate a more geographically, culturally, and socially diverse array of perspectives.” And this seems to be going well: according to the authors, the framework has ‘influenced’ the OECD’s Recommendations of the Council on Artificial Intelligence. The suggestion made here is that the framework presented is of such a high quality that it was valuable enough to be included in a document composed by a large and important international organization. What this exactly means in practice, how this framework was able to make this happen, and what this implies for the numerous guidelines that were not included, is not discussed.^6^

I take Floridi and Cowls’ paper exemplary for a particular way of doing ethics where the construction of clearly defined and coherent ethical frameworks is of prime importance.^7^ Researchers identify various reasons for doing ethics in this way. From within a ‘data ethics’ perspective, wherein the practice of doing ethics is connected with regulation and law, ‘hard ethics’ are concerned with values, responsibilities, rights, and duties (Floridi, 2018). The construal of ethical principles is therefore directly relevant and related to the writing of regulation and legislation.^8^ Forms of ‘soft ethics’ provide guidance “over and beyond existing regulation,” and presuppose the legitimacy of existing legal frameworks and human rights (Floridi, 2018). From this perspective, the convergence of many of the AI ethics lists and guidelines is indicative of the gradual identification of what is considered *good* practice (Taddeo & Floridi, 2018).^9^

The second step of this type of ethics is the translation of principles into practice, as a means to remove the gaps and frictions separating them (Floridi et al., 2018; Morley et al., 2020; Stix, 2021). When translations are successful, these guidelines and frameworks could, for instance, be used to promote internal organizational change by helping individuals act better, set behavioral standards in- and outside of the organization, or promote (moral) organizational leadership (Jobin et al., 2019; Metcalf et al., 2019; Schiff et al., 2020). The actual enforcement of principles does not always have to be their aim. From an academic point of view, ethical frameworks can be understood as scientific output, illustrating the growth of our knowledge about what is good and right.

Whether these promises come true is, however, debated. Thilo Hagendorff, for instance, cites the results of a controlled study that shows the complete lack of effect of ethical guidelines on the behavior of software engineers (Hagendorff, 2020, p. 108). But regardless of whether this result can be extrapolated to other contexts and principles get ‘lost in translations,’^10^ the fact that especially commercial actors participate more in the production of ethical guidelines than in the enforcing of ethical guidance does makes these lists more susceptible to be used as paper tigers, rather than attempts to improve policy and practice. And just like tech developers should, arguably, be held accountable (if not responsible) for the problematic usage of their technical products, do I think that producers of lists and guidelines have a responsibility for the kind of (mis)use of their academic products by commercial actors. If data scientists have a ‘politics’ because they are capable of influencing the world, data ethical practices are equally political (Green, 2018).^11^ Moreover,

(…) detached, universal rules have a habit of becoming dissociated from the original moral urgency that led to their drafting. Ethics codes that have substantial effect on institutions can result in an infrastructure whose effective management demands more attention than the actual ethical commitments underlying the code. It is important to recognize the risk that the universalism and detachment favored by philosophical ethics can lead away from facing the most concrete ethical challenges and instead leave us with routinized obedience to an infrastructure. (Metcalf, 2014, p. 7).^12^

For these and other reasons,^13^ authors have been putting forward alternative approaches as means to combat problematic techno-political structures. Examples are the aforementioned STS, global data justice, and human rights approaches. Others have tried to slow down the move away from ethics as a valuable tool or practice. Ethics could, the argument goes, still be of value in an educational or possibly even organizational context (Bates et al., 2020; Mittelstadt, 2019; Moore, 2020; Taebi et al., 2019). The problem is that it is not clear what then ethics’ contribution would, could, or ought to be in combating ethics washing in the tech industry. In the next section, I put forward an explanation for ethics’ limited contribution in this context, based on criticism raised by political philosophers on dominant strands of moral and political philosophy. I thus take this criticism to be also helpful in making sense of the construction of ethical guidelines in the tech sector.

## From Theory to Practice; from *Phronesis *to *Episteme*

Top-down attempts to define ‘good principles’ and eventually arrive at ‘good practices’ (Floridi & Cowls, 2019) correspond to what Raymond Geuss calls ‘ethics first’ approaches, and what Albert R. Jonsen and Stephan Toulmin would label as ‘theoretical,’ rather than ‘practical’ accounts of ethics. Jonsen and Toulmin’s work on casuistry supplies us with both an historical explanation of why there is in general a predominance towards the construction of ethical frameworks and theories (and why this was not popular for the biggest chunk of human history), and argue for a revival of a forgotten and discredited mode of ethical practice: casuistry. I first quickly summarize their argument before coming back to that of Geuss in Sect. 4, who allows us to critique the practice of casuistry as well.

In the 1970s, Jonsen and Toulmin were members of the ‘National Commission for the Protection of Human Subjects of Biomedical and Behavioral Research,’ which was set up after the controversial Roe v. Wade decision by the Supreme Court (Jonsen & Toulmin, 1988, pp. 16–17). The commissions’ job was to investigate all sorts of ethical issues related to the research on vulnerable human beings and consisted of experts with a wide variety of backgrounds. One might expect that discussing ethical issues and dilemmas with individuals with totally different (personal, religious, educational) upbringing might not be the best decision if one wants to come to agreements and advice. Such a discussion would probably end up in a verbal trench war where every commissioner hides behind her or his moral principles. To Jonsen and Toulmin’s surprise, this was far from the case: the commission was usually capable of finding agreement on what to do when they discussed specific issues in practice. Agreement was not based on the construction of consensus on the level of principles (because this was absent), but on a “shared perspective on what was specifically at stake in particular kinds of human situations” (Jonsen & Toulmin, 1988, p. 18). Only at the moment the commission left the realm of practical affairs and entered that of moral reasoning did differences of opinion start to emerge. For Jonsen and Toulmin, their experiences as members of the National Commission were indicative for two different understandings of ethics and morality. The first one emphasizes the importance of finding universally valid principles. The other aims to arrive at an understanding of ethical issues and their context. The problem was that the approach stimulating argumentative deadlock seemed to be the most widespread, if not dominant, understanding of ethics up until today.^14^

This difference between a more ‘theoretical’ or ‘principle’-oriented account of ethics, and a ‘practical’ and practice-oriented one can be traced back at least 2500 years ago when Aristotle distinguished between two modes of knowing (*episteme* and *phronesis*) and placed the practice of ethics in the second category, and by doing so made it effectively a practical rather than theoretical affair (Jonsen & Toulmin, 1988, Chapter 1). In contrast to *episteme*—whose statements have an idealized, atemporal, and necessary character—practicing *phronesis* is concrete, temporal, and presumptive. *Phronesis* is the art of judging what to do in concrete situations, without assuming that the judgments will hold for everyone, everywhere, and every time. Aristotle’s emphasis on the practical issue-based character of moral practice, where *phronesis* was to be found of greater importance that the ‘correct’ label (whether ‘ethical,’ ‘legal,’ or ‘political’) attached to the issue in question, eventually managed to influence a branch of religious problem-solving called casuistry. Casuistry involved the attempt to make sense of moral issues in their practical context by applying a mixture of case-study analysis, comparison with similar cases, and (religious) principles. Though the casuists never formalized their methods in a theory of some kind, Jonsen and Toulmin were able to distill from the cases they studied several common features that they summarized in the following way. Casuistry.

is the analysis of moral issues, using procedures of reasoning based on paradigms and analogies, leading to the formulation of expert opinions about the existence and stringency of particular moral obligations, framed in terms of rules or maxims that are general but not universal or invariable, since they hold good with certainty only in the typical conditions of the agent and circumstances of action (Jonsen & Toulmin, 1988, p. 257).

Like their medical colleagues, those practicing casuistry required a degree of *phronesis* to be able to judge whether their analysis was appropriate. This judgment did not revolve only around the question what rules should be considered and what could be learned from them, but also to what extent the application of the rules within or across several cases was fair and appropriate. This required in-depth knowledge about the circumstances out of which the issue presented itself, which also informed one’s understanding and application of the relevant (moral) principles applied.

Not surprisingly, some found the casuistic method to be lax and disrespectful towards the important moral beliefs of Christian faith. Blaise Pascal, the famous mathematician, accused the casuists of being unfaithful because as they “(…) pursued their analysis of the moral life into more and more detailed cases, they seemed to move further and further away from the clear light of these beliefs.” (Jonsen & Toulmin, 1988, p. 238). The casuists tried to connect the strong religious imperatives of their times with the worldly demands they encountered through the continuous adjustments of these principles, and where both being accused of being lenient “towards the claims of morality” or of contradicting the maxims and principles they (personally) hold so dear (Jonsen & Toulmin, 1988, p. 259). Pascal’s criticism set into motion a move towards the attempt to further ground and stabilize ethical theory, at the cost of improving its practice. Next to these theoretical worries about casuist moral practice, the slow deterioration of the Catholic Church further complicated the lives of professional casuists. What is appropriate casuistry is not to be distilled from the arguments put forward by individual professionals alone, but intimately relates to the institutional culture of which they are part—just like in medicine. And the importance of this explanatory factor for the deterioration of the practice of casuistry is not to be overstated. Next to theoretical and methodological validity, a proper functioning moral practice like casuistry also needs a secure institutional embedding. Medical ethics always had such an environment, which helped practitioners to determine whether they were doing well or not. Moral casuistry too—up until the moment this was broken down when religion slowly started to lose its dominance and ‘Enlightenment’ thinkers (e.g., Galileo, Descartes, Newton, Grotius) started to look for more secure scientific, and rhetorically convincing foundations, which set the standard for moral-legal ones (Jonsen & Toulmin, 1988, pp. 275–276, 331).

Moving forward two centuries, Jonsen and Toulmin argue that the move away from practical affairs into moral theory was still taking place in the twentieth century. Moral philosophers in Britain and the USA (e.g., Moore, Perry, Ross, Stevenson, Rawls, Hare) were only interested in practical issues to the extent that these could serve as “(…) new ways of testing the intellectual merits of rival theoretical positions” (Jonsen & Toulmin, 1988, p. 281). Though there were some exceptions (James, Dewey), moral philosophy was often considered to be of theoretical nature—this in complete contradistinction with how Aristotle understood ethics.^15^

Following Jonsen and Toulmin’s analysis, it is no surprise that the kind of data ethics undergirding our example of an academic ethical AI framework is also positioned as opposed to a form of computer ethics focusing on practitioners, prone to “(…) the spreading of ad hoc or casuistic approaches to ethical problems” (Floridi & Sanders, 2002, p. 1).^16^ Casuist forms of computer ethics, for Floridi and Sanders, lack a thorough conceptual foundation and because of that are at risk of presenting conservative and dogmatic answers to the question of what needs to be done, and of presenting ethical problems as simple. In contrast to such a professional computer ethics, a more ‘mature’ theory is needed that explains the meaning of information and digital ethics.^17^ And such a digital ethics “(…) studies and evaluates moral problems relating to *data* and *information* (…), *algorithms* (…), and corresponding *practices* and *infrastructures* (…) in order to formulate and support morally good decisions (…)” (Floridi, 2018, pp. 3–4). The overabundance of guidelines and frameworks, however, suggests that the identification of moral problems and moral principles (as *episteme*) tips the scale at the cost of more practice-oriented work (as in *phronesis*).

## The Problem of Ethics (Washing): A Political Proposal

Jonsen and Toulmin suggested that the strong focus on the truth or strength of ethical principles forecloses a lot of different ways wherein ethics or morality could be practiced and be meaningful to human life (Jonsen & Toulmin, 1988, p. 302). I suggested that the dominant form of AI ethics indicates that their conclusion is also valid to that discussion.^18^ Throughout various essays Cambridge-based philosopher Raymond Geuss takes a similar issue with this limiting, limited, but nevertheless dominant view on ethics to be found in Western academia. Already the question ‘what ought I do’ seems to assume that there is some determinate answer to be found and that that is also the most important question one should ask and also answer if your business card reads ‘ethicist,’ ‘philosopher,’ or ‘Chief Ethical and Humane Use Officer’ (Geuss, 2005a; Kinstler, 2020). In this section, I draw from Geuss to complicate the picture of ethics sketched by Jonsen and Toulmin. Jonsen and Toulmin argued for a revival of a moral practice wherein the judgment of professionals about particular issues within their context would help in determining what to do. I first explain what kind of ethics is implicit when one aims to help someone finding out what to do. I then deal with the assumption that specific individuals are burdened with the task of helping people answer that question within the context of ethics washing in the tech industry. I argue thirdly that a more broadly conceived understanding of ethics, combined with a more open understanding of who and where does such an ‘ethics,’ is more appropriate to our context than casuist (and principle-based) approaches.

Let’s start with the idea that ethics is about finding out ‘what I should do’. This question seems to be present in many of the published ethical guidelines and principles. But formulating this question is as old as moral philosophy itself and goes back to the times when Aristotle laid down the first bricks of the building that would eventually grow into medieval casuistry (to be broken down some centuries later). And the assumption that any individual at every moment would and should be able to ask the question what to do, and also find an answer to it (often grounded in some external authority like god or reason), is still the most dominant understanding of ethics to be found in the Western world (Geuss, 2005a, pp. 44–45).

The first thing one can learn from Geuss’ reading of several Central-European thinkers (e.g., Hegel, Nietzsche, Adorno, Heidegger, Kierkegaard) is that the ethical question assumes the existence of an ‘Ego’ (Nietzsche) with a limited number of possible courses of action of which he can freely choose (Geuss, 2005a, pp. 54–55).^19^ Nietzsche (in Geuss’ reading) rejects this view on human decision-making because it presumes a false choice between free and unfree decisions. Instead of assuming that people are free to decide to engage in a morally good or bad activity, concrete^20^ human beings have different wills or interests that have an influence on whether they want to answer, let alone ask, the ethical question in the first place. An individual’s capacity should thus be taken into consideration when thinking about whether ethics is a valuable addition to a particular individual’s life, or not. For some mighty individuals, answering the question what one is ought to do might be one of the last things they think about when living their life. Others, by contrast, simply lack the means, capacities, or time to engage in such reflections (Wright, 2021, p. 126).

This also relates to a second criticism levied by some of the thinkers discussed by Geuss. Both Hegel, Nietzsche, and Heidegger damned the idea that specific individuals like philosophers or casuists are the most capable of dealing with the question ‘what ought I do?’. Better ask the experts in the respective domains out of which an issue arises, than relying on a philosopher and his knowledge about Theory, the World, and the Good (Geuss, 2005a, pp. 51–59).^21^ This criticism connects well to a comment by danah boyd and Solon Barocas that it is too easy to criticize the tech experts for not respecting important values of some sort without having any understanding of how the technology in question is being construed in practice (boyd & Barocas, 2017).^22^ Geuss, and boyd and Barocas hint here at an important and complicated question to be asked within the context of this essay: who should do ethics? I take these authors’ commentary to imply that it would be worrisome to do ethics from far away and/or in isolation from the practice concerned.^23^ This, I add, for three reasons. First, good criticism and *phronesis* require a thorough understanding of the activity being judged. Proper judgment depends on a thorough analysis of the problem (Geuss, 2010).^24^ Second, the legitimacy or value of one’s judgment depends on how it relates to the norms, values, and sensibilities that structure the practice in question (Bennett, 2001, pp. 156–157). Your judgment, and its implicit moral presuppositions, should have some traction in the context to which it is directed, to be able to make sense. Lastly, the quality of your intervention also depends on its uptake in practice (Krause, 2015). Evaluating and reflecting from afar, would not do much good. There are thus epistemological, moral, and practical reasons to be found that put into question the idea that individual philosophers are best equipped to deal with the question what someone should do. I will come back to this below.

But the question is not only if philosophers ought to be answering the ethical question, but whether individuals *überhaupt* are the kind of entities that should attempt to change their reality by helping others or themselves act ethically. Especially Theodor Adorno was pessimistic about this. Individual attempts to instantiate social change, whether by philosophers or Chief Ethical and Humane Use Officers,

(…) may be morally praiseworthy but cannot be more than a palliative measure, and will certainly not be sufficient to deal with the deep-rooted and systematic social and economic sources of suffering.^25^

One cannot, according to Adorno, reasonably hope that individuals have the capacity to change the kind of systematic injustices we as inhabitants of the modern world face.^26^ Especially not, Geuss adds, when one is merely looking into what one ought to do. These and some other arguments lead Geuss to argue for a broadening of our understanding of what ethics is or could be to avoid restricting oneself to only look at a limited set of ‘ethical’ issues, topics, and methods out of which an individual should choose.^27^ The arts, history, and politics are some of the domains to which Geuss draws attention. And that is also something we should do when dealing with the issue at stake: ethics washing.^28^

Fortunately, in later work, Geuss gives a more elaborate answer to the question what these arguments imply for political philosophy and, as I suggest, our politicized conception of data ethics. Geuss rejects here understandings of philosophy and ethics that start with the formulation of the most ideal moral situation, and after that apply that to practice (Geuss, 2008, p. 6). ‘Ethics first’ approaches seem to assume that ethics is a discipline one can study separate from empirical reality and scientific approaches like history, sociology, and economics, and that it is possible to formulate a limited number of moral principles that apply to and prescribe the actions of individual human beings. Broadly along the same lines as the previously discussed practically minded casuists,^29^ Geuss argues that political philosophers should start with human practices and investigate action rather than beliefs, take into account history into their analysis, and acknowledge the fact that acting politically requires the skill to judge whether some course of action is appropriate in a certain context or not (Geuss, 2008, pp. 9–16). Theories are unlikely, as Geuss argues, to help one to make such *political* judgments. And politics is in the end, due to the interminglement of academia and industry, and whether they like it or not, the activity ethicists are engaged in (and what they also *should* explicitly be engaged in).

Arriving at a proper understanding of the political context you study is thus to be the most important first step doing ‘realist’ political theory.^30^ And instead of principles, questions are the way to go. Geuss identifies three broadly formulated questions which function as starting points when acting politically (Geuss, 2008, pp. 23–36).^31^ These are (in my own words):

- Who does or could do what to whom for whose benefit?
- What is the right moment on which I or we should commence this or that action?
- What are the ‘legitimatory mechanisms’ in place and how do they relate to the proposed course of action?

Asking yourself these three questions forces you to make sense of the complicated social-political context wherein one is thrown, its various temporal characteristics (past, present, future) while also considering the relevant norms and values that have an impact on why some actors do what they do, or not, and how you should relate to them. Doing so is especially valuable in a context where the main units of analysis—‘AI’; ‘algorithms’; ‘machine learning’—are highly ambiguous. While some scholars, for instance, understand algorithms as ‘technicalities,’ others argue that one should study these as constantly changing cultural objects.^32^ Assuming it is important to understand what one’s judging when practicing ethics, paying careful attention to how particular technologies, norms, human beings, and organizations interrelate is crucial. In the light of Geuss’ own discussion of the limitations of conventional ethics, I add one more question to be asked:

• With whom should I engage in the process of question and answering?^33^

Since one’s understanding of the social-political context is increased when one interacts with this context itself, and one’s action is probably also more effective and legitimate when one acts not alone, it is crucial to question the collective character of one’s intervention. Especially this is often ignored in list-based approaches (Metcalf, 2014).

There are thus three intimately related but different advantages of Geuss’ more ‘realistic’ political philosophy noticeable. By starting with the attempt to understand a political practice first, you (a) will improve the epistemological character of one’s evaluation or critique. You are better capable of understanding why not only something is ‘good’ or ‘bad’ in a particular (cultural) context, but also how the ‘good’ and the ‘bad’ are constituted in the practice, and during one’s interacting *with* the practice. Especially this feature sets a practice-oriented approach apart from approaches that presuppose the relevance of particular norms *prior* to engaging in their analysis. You (b) will also improve the normative character of the critique when the practice, its history and future, and ‘legitimizing mechanisms,’ are properly studied because you will be able to explain better why your evaluation has moral relevance in that particular context (if any). A and b combined will increase (c), the practical uptake of one’s intervention. One example to illustrate this is the act of criticizing Amazon for the damaging impact of their ‘Echo dots’ on the environment.^34^ It is relatively easy to argue that Amazon should stop their activities and adopt a more friendly or even ‘just’ attitude towards the environment. But even if you as individual would be able to convince Amazon to change their business model, the fact that Amazon is part of a global capitalist structure will not solve the problem because a Google or some other company will take Amazon’s place. This example pertains to show that it matters hugely how critique is presented and organized, and how in general the criticism itself is related to the criticized practice. Should your intervention have an individual or collective character? Should it be internal or external to the practice studied? Is its scope local or global? Are you interested in instantiating reform (criticize BP) or revolution (fight capitalism)?^35^ When you want to change the tech industry, answers to these and other questions should be looked for and be incorporated and explicated in one’s own analysis.^36^ And questions like these only pop up when you start by asking questions—like, for instance, those four listed above—and not by formulating lists of principles and guidelines.

## Contemplation, Action, and Empirical Research

One dominant way of answering the ethical question is thus the deontology-like method of writing ethical guidelines and lists. One reason for practicing ethics in this way is the idea that the identification of moral principles will trickle up into legislation helping to enforce morally good behavior. Such an approach is to be preferred, the argument goes, over more context or casuist alternatives because these are less capable of identifying what is good and enforcing actors’ behavior in a clear and consistent manner.^37^ Theorizing about what is morally good, thus in the end, after a complicated process of translating principles into practice, helps bringing about this good in practice.

The implicit ethical question presupposed in such reasoning, however, can be criticized for its individualistic character. Not only is the idea that one should make *decisions* based on limited conceptions of individuality, one can also question the presumption that a specific category of individuals is best equipped to answer them, and moreover, whether the implicit focus on individual behavior is appropriate. I argued for the value of starting engagement with techno-political practices in *practice*, rather than in deontological theory, by asking four broadly formulated questions. These questions form the basis of a more practice and empirically minded approach towards the studying of (dubious) techno-political practices.

The notion of practice is crucial here. Practices can be described as “self-organizing and self-investigating phenomena” and what kind of norms and meanings they enact is something to be studied rather than presupposed (Lynch, 2001, p. 140). Moreover, identifying and imposing moral principles from afar might amount to the unwarranted imposition of the good, the right, or the just (Boddington, 2020, p. 127). For reasons like these, scholars from a variety of disciplines have been arguing for more practice rather than theory-focused forms of research. Political theorists presented forms of public, ethnographic, or ‘realist’ ‘theories,’^38^ ‘praxiographers’ emphasized the importance of—surprise!—‘practice,’^39^ and others argued for ‘empirical philosophy,’^40^ while science and technology scholars have called for more attention for more experimental forms of political ontology (Marres, 2013) and sociotechnical analyses (Green, 2021). Especially Marres’ work is helpful here because it explicates what proper *political* judgment amounts to. Rather than analyzing if and how technologies have consequences for democracy and politics, and what this than means for our moral evaluation of them, Marres argues that the analysts’ challenge is to redescribe *how* technologies *transform* practices of politics and democracy, and the norms they constitute, themselves. Algorithmic decision-making, for instance, does not merely affect how promptly a citizen receives her decision (and whether this is good, bad, accountable, or transparent), but also has implications for how the process of decision-making, and hence the ‘decision,’ should be understood.

The thread connecting these different ‘turns to practice’ seems to be the idea that sociotechnical assemblages cannot be understood, governed, and criticized without a proper grasp of their functioning in practice, and that such a grasp cannot be attained from a distance, or ‘in theory’.^41^ Just like Bernard Williams worried about the ‘limits’ of philosophy that reduces the ethical question to one of rights and duties (Williams, 2010), we should worry about attempts to reduce good tech development to one of the translation and implementation of moral truths.

## From Principles to Exemplars

In the epigraph above, Wittgenstein suggested that there are limits to how rules could structure practices because of the self-sufficiency of practices, and that one needs *examples* for this as well. The presentation of examples can be understood *not* as mere illustration of an important moral value, but as an independently valuable ‘tool’ in the analysts’ toolbox. Part of the reason why it is important to study both past, present, and future of the context wherein a particular moral issue might (or might not) play has to do with the fact that few issues are so new, innovative, and revolutionary that we really have nothing else to do than to come up with a new guideline, paper, or book explaining what to do.^42^ Revolutions are, by definition, rare.

Being reminded about especially the historical predecessors to the kind of issue you are interested in does not only contribute to the fabrication of the kind of collective memories that are important for the continuation or disintegration of communities and institutions—both are crucial within the context of ethics washing (Mittelstadt, 2019). Doing such research as part of your ethical–political judgment also supplies you with exemplars that you could follow (or not).

In a forthcoming chapter, David Owen explains in what way the work of James Tully—a ‘realist’ colleague of Geuss (Honig & Stears, 2014)—differs from ideal and principle-oriented political philosophy (Owen, Forthcoming). Owen argues that Tully (and in my reading also Geuss) enacts a form of political philosophy where not principles but ‘exemplars’ are to be sought for. The difference is that.

(…) principles aim to tell us what, as an agent, we ought to do or be, exemplars manifest what it is to be an agent characterized by such doing or being (or, to put it another way, principles tell us another world is possible, exemplars show us that another world is actual). Second, whereas a principle articulates norms in their generality, an exemplar discloses norms through its individuality. (Owen, Forthcoming)

Authors narrating exemplars thus draw from the past, present, or future to show us that the world can indeed be different, and by focusing on how in other circumstances the world was and is indeed different, they allow those concerned to learn from these stories. The kind of learning involved is not that of the imitation of the application of a rule (e.g., the check-box approach to ethics and law), but is meant to give guidance through ‘following’ (Owen, Forthcoming). Or, in other words, stories about individuals or collectives that attempt to bring about change to tech companies will help elucidating the kind of questions, worries, and challenges such actions might generate. What can we learn, for instance, from Floridi and Cowls’ success in composing and communicating their list of principles? How did such a process go? How did they manage to get into the field? And how does being the head of a prestigious research institute effect such an activity?

A good example of academic work done along the lines of the argument presented so far is Catherine D’Ignazio and Lauren F. Klein’s *Data Feminism*. Next to its embrace of a ‘power challenging’ approach operationalized in the form of a dismissal of status quo-affirming concepts like ‘ethics,’ ‘fairness,’ ‘accountability,’ and ‘transparency’ (D’Ignazio & Klein, 2020, pp. 59–64) is their book also packed with stories narrating the attempts of collectives to challenge techno-mediated oppressive structures. From Joy Buolamwini’s analysis of the racist features of face-analysis software, to María Salguero’s mapping of feminicide in Mexico, to the massive ‘Google walkout’ at the end of 2018, D’Ignazio and Klein’s inclusion of these stories informs readers about how and why individuals and collectives tried to deal with big tech’s oppressive characteristics. These stories are not mere illustrations of the quality of D’Ignazio and Klein’s argument, or smaller pieces of the jigsaw puzzle that when finished would be able to supply us with an answer to the ethical question. “What is most important is not that we all share the same starting [or end^43^] point, but rather that we nurture all these emerging ecosystems and build links between them (D’Ignazio & Klein, 2020, p. 214).”

## From Ethics to Politics, or What Is Left of Ethics?

Throughout this essay I argued for the importance of a more-than-theoretical ethical approach towards the growing influence of (commercial) technology on society, by emphasizing the importance of empirically informed analysis of techno-political practices. Ethics in this framing becomes more than the identification of important values, is an inherently collective and interactive endeavor, and requires the acknowledging of the conflictual character of doing political work. In a recent ‘tweet,’ political philosopher Annette Zimmermann posted an adjusted version of the ‘types of scientific paper’ cartoon by XDCD, wherein she identified various ‘types of AI ethics’ (Zimmermann, 2021). The argument presented so far in this essay fits comfortably within the ‘Why ethics (and philosophy more broadly) can’t possibly address questions of power’-type, and also has close affinities with other recent calls for more politicized, activist, feminist, and power hierarchy-sensitive calls to action (Barabas et al., 2020; Beraldo & Milan, 2019; D’Ignazio & Klein, 2020; Green, 2018; Ruppert et al., 2017; Sloane, 2019; Taylor & Dencik, 2020). From a variety of academic backgrounds (e.g., STS, feminist theory, anthropology, social movement studies), scholars thus try to make sense of the problematic appropriation of ethics by the tech sector. What remained undeveloped so far, ironically, is substantive philosophical reflection onto why the formulation of principles and guidelines is so dominant, what this tells us about how ethics is (implicitly) being understood in the ‘Western’ (tech) world.^44^ I hope to contribute to this discussion by providing a historically informed reformulation of ethics, where the asking of questions, empirical analysis, and the usage of exemplars has priority over the construction of ethical theories, principles, or guidelines. The question remaining is whether the label ‘ethics’ is still appropriate for the ‘repoliticized ethics’ that I am arguing for.

The answer to this question is negative. When Wagner and Delacroix made reference to both Mouffe, Williams, and Geuss when arguing for a ‘supportive interface’ between answers to the ethical question and regulation, they seem to have overemphasized the constructive character of this relationship (Delacroix & Wagner, 2021). For them, just like in Floridi’s digital ethics, and Seger’s ‘principled’ approach (Seger, 2022), ethics and legislation are important components of a regulatory apparatus. What for Mouffe, Williams, Geuss, and other scholars is also, and maybe more, important are the agonistic elements of making ethical–political judgments.^45^ At least two claims could be distilled from the works of these authors. First, there are differences, tensions, and incompatibilities between individuals, worldviews, societies, and theories which cannot all be completely removed through processes of deliberation, theorizing, or consensus forming. Second, there is also value in *not* attempting to remove all these differences, and to also support activities that disrupt, contest, and say ‘no’ to ossified modes of thinking or doing.^46^ Extrapolating these thoughts to our AI ethics discussion, I argue that the relationship between (academic) ethics, especially in its popular deontological form and political practice, and legal/corporate practice, could and should not only be understood in terms of support, consensus, and recommendations, and be opened to allow room for contentious alternatives.^47^

For reasons like these, one should be wary of Floridi’s explicit rejection of ‘solution-less’ types of ethics that do not provide ‘acceptable recommendations’ because that unduly limits the kind of practices human beings can engage in in response to the excessive influence of technologies on their lives (Floridi, 2018, p. 7). Accepting such restrictive definitions of ‘digital ethics’ “(…) is to allow the existing social formation to dictate the terms on which it can be criticized, and to allow it to impose a theoretically unwarranted burden of positive proof on any potential critic” (Geuss, 2008, p. 96). Moreover, the idea that it is always worthwhile to try to implement and translate ethics as answer to the ethical question in commercial practices should be reformulated as a question in itself rather than as aim everyone should strive towards.^48^ Acting politically, then, is a collective and potentially contentious activity. While I do not deny that some forms of ethics can also be characterized as such, I suggest that labeling such practices as ‘politics’ rather than ‘ethics’ makes them less susceptible to be washed away in the PR machinery of the tech sector. Doing so, secondly, also emphasizes their practical, bottom-up character, which lastly also invites non-academics to the discussion. You do not have to be an expert in Kant, Bentham, or Aristotle to be able to participate in AI politics.

For these reasons, I argue, quite unoriginally, for a strengthening of forms of ‘data politics’ to tip the scale in favor of more practice and politics-oriented forms of (academic) research, as a means to even out the excessive focus on ethics in its principle and theory-casted form.^49^ For the individual ‘ethicists’ herself, this reorientation will amount to transforming from an ‘universal intellectual,’ interested in “society as a whole and what is ‘just and true for all,’” to a ‘specific intellectual’ analyzing “games of truth, relations of power and ethics in the practical systems in which he or she is engaged, their historical formation and possibilities of modification.”^50^ The move from theory to practice at the level of the *practice of AI/digital ethics*, I argue, is thus accompanied by a move from universality to specificity at the level of *the individual researcher*. ‘Ethicists,’ whether in academia or elsewhere, should ask for new business cards, replace ‘ethical’ by ‘politics,’ and their guidelines with an in-depth analysis and a good story or two.

## Outro

I want to end this essay with two comments. First, an important question my argument touched upon was concerned with the meaning of ethical–political judgments, and how this is reflected in academic products. I argued for a certain reflexivity concerning the establishment and embeddedness of the theories, guidelines, and recommendations produced by ethicists and philosophers. It matters how questions and arguments are presented, by whom, to whom, when, and where, and ignoring these components of one’s ethical–political judgment does not help in understanding how particular types of academic work do what they do. Making such characteristics more explicit helps to analyze and value ethicists’ contributions, and is thus conducive to empirical scrutiny of their work.^51^ Second, my argument’s primary aim was to make a case for the need to diversify the types of ethics to be found by pointing at some downsides of theory-oriented, list-producing, and regulation-directed approaches. Through showing that this dominant way of doing ethics had not been around for a large part of human history, I open up conceptual room for other ethical repertoires and by doing so also contribute to the defusing of worries that collective, contextual, or more political forms of action might not fit or belong in current tech practices.^52^ At this moment, indeed, this might be not how tech development in general goes,^53^ but that does not mean that change is inevitable, and that we should accept the prevalent mode of doing and understanding ethics. Ethics, in other words, does not necessarily have to be the individualistic and abstract academic practice it often is, and I urge readers into reflecting about how they position themselves as ethicists, what kind of role their academic products have, what it means to engage in what kind of evaluations, in what way they acknowledge their situatedness as researchers (especially vis-à-vis industry), and last but not least what kind of ‘theory of change’ implicitly motivates their work. Do you think that being ‘close’ to the tech industry helps instantiating change, or would it be better to stand further away from the target of one’s criticism (Sætra et al., 2021)? Based on the argument developed throughout this essay, the kind of ethical research currently popular is arguably too close or at least too easily subsumed into industry, which does seem to make it more difficult for those interested in change situated afar (Sætra et al., 2021). Acknowledging this is the first step in broadening the ethical repertoires, which will allow practice and contentious-minded approaches to make a stronger case for their viability, to in the end *also* get that seat at the negotiation table (reform), or the capacity to demolish it altogether (revolution).

## Data Availability

Not applicable.

## Footnotes

1 And not *necessarily* the one practiced by data scientists or engineers.

2 Or by understanding ethics as a domain independent from science and politics (Sætra & Fosch-Villaronga, 2021).

3 See Williams (2010). See, within the context of AI ethics, Ananny (2016, p. 94); Delacroix and Wagner (2021, p. 2).

4 See e.g. https://inventory.algorithmwatch.org/about.

5 See also, Floridi (2019).

6 It is also unclear how Floridi and Cowls’ reading of the guidelines used relates to the various motivates attributed to the writing of such guidelines by various actors. Does it make a difference for the (moral?) quality of such a list when a particular document’s primary function is a company’s self-promotion?

7 I hereby thus presuppose the existence of interactions and mutual influence between academic instances of data ethics, and their commercial counterparts. On the mutual *dependencies* existing between tech ethics and big tech, see Hu (2021). Taylor and Dencik identified several other ‘nodes’ of the data ethics network (Taylor & Dencik, 2020). See also Green et al., who explained why it is relevant to study those guidelines in the following way: “we suggest these statements represent the transformation of ethics and design into *discourses about* ethics and design. And as with discourse broadly, these statements legitimate (delegitimize) certain practices, providing “answers to the spoken or unspoken questions ‘Why should we do this’ or ‘Why should we do this in this way?’” (italics in original) (Greene et al., 2019, p. 2124).

8 See also Delacroix and Wagner (2021); Seger (2022).

9 But see also Rudschies et al. (2020).

10 On this see also Mittelstadt (2019).

11 As said above, neat distinctions between the domain of ethics and that of politics are hard to sustain both empirically and normatively. On the politics of (social) science, see e.g. Law and Urry (2004).

12 More downsides are listed by Boddington (Boddington, 2020, pp. 129–130).

13 Lobbying and funding structures could also affect the kind of research being done by tech scholars, though that is always hard to prove. See e.g. Clarke et al. (2021).

14 Though Jonsen and Toulmin wrote in the 1980s, I take more recent works by ‘political realist’ as indications that their argument for moral philosopher generally still makes sense.

15 The situation for political philosophy, specifically, was not that different. See e.g. Forrester (2019, Chapter 8); Freeden (2018, pp. 348–351).

16 A recent study of papers published in two influential AI/computer science conferences “shows that there is a great tendency for abstract discussions of such topics devoid of structural and social factors and specific and potential harms” (Birhane et al., 2022, p. 13).

17 See e.g. Floridi and Taddeo (2016).

18 Green points at two other sectors—ethics in science and corporate ethics—where similar problems with respect to the usage and abuse of ethics are present (Green, 2021, pp. 216–218). This supports my argument that the problems present in AI ethics can be explained as symptoms of a more general way of understanding ethics in (Western) academia.

19 From a different angle, Annemarie Mol also argues for the need to move beyond a ‘politics of choice’ (Mol, 2014, pp. 178–179). See also Amoore (2020, p. 66).

20 See Benhabib (1992).

21 See for a similar approach Tully (2008).

22 See also Lee et al. (2020).

23 Compare “Ethics thus operates at a maximum distance from the practices it actually seeks to govern” (Hagendorff, 2020, p. 112).

24 And moreover, an embeddedness in the practice one’s judging. On this, see Fossen (2021).

25 As quoted in Geuss (2005b, p. 113).

26 While the Holocaust was in the background of Adorno’s writing, forms of ‘surveillance capitalism,’ ‘data colonialism,’ and a ‘surveillance culture’ could be named as the structural features that currently position “the individual at the losing end of the equation.” (Beraldo & Milan, 2019, p. 4).

27 Compare the long lists of values and ethical issues related to AI, which seem to suggest that a reader can *choose* not only which value he could ‘use,’ but also what ethical problem could be relevant to his context.

28 See the other essays in the several volumes quoted, and specifically Geuss (2005c). See for recent similar arguments three blog posts published at the Ada Lovelace Institute (Powell, 2021; Tasioulas, 2021; Vallor, 2021).

29 But different due to his dismissal of the relevance of ‘the ethical question.’.

30 The other four phases identified by Geuss are evaluating, orientation, conceptual innovation, and ideology analysis and criticism. Though these are important components of Geuss’ realist political philosophy, I take them to be less relevant for the topic at hand. On realist political philosophy, see e.g. Prinz (2019); Rossi (2019).

31 See, for a similar argument within the context of AI ethics and robotics, Keay (2020).

32 See this illustrative discussion in Dourish (2016); Seaver (2017).

33 This addition amounts to an internal criticism of Geuss’ latent existentialism. I thank Joel Anderson for this suggestion.

34 I adjusted an example Geuss used in Geuss (2014, pp. 78–79), with the help of Crawford and Joler (2018).

35 Ben Green notes that data scientists have a more reformist rather than revolutionary attitude towards ethics (Green, 2018, pp. 25–26). On such ‘dilemmas,’ see Sætra et al. (2021).

36 You cannot rely solely on one’s disciplinary background to only do ‘law,’ ‘ethics,’ ‘environment,’ or ‘privacy’ because the appropriate tool one should pick depends on the character of the practice in question. Of course, what the practice is depends on the glasses one’s wearing, which are tainted by one’s background. There is a complicated interaction between practice-understanding, problem-definition, and methodology, though I suspect that the dominant research chronology is still background → solution → application → practice – which I thus criticize in this essay.

37 The criticism sometimes raised against ‘realist’ political theorists that they reify a dichotomy between ‘real’ and ‘ideal’ forms of theorizing is thus equally applicable to (ideal) forms of data ethics.

38 See e.g. Herzog and Zacka (2017); Sleat (2018); Tully (2008).

39 See e.g. Lynch (2001); Mol (2014).

40 See e.g. Dubbeld (2005); Mol (1994).

41 Paul B. De Laat’s thorough attempt to diffuse the ethics washing critique based on commercial actor’s self-published material illustrates this (de Laat, 2021). As he explains himself, it is not possible to say much about companies’ actual adherence to ethical norms and their implementation based on material found online.

42 Though academic practice often suggests otherwise. See also Uricchio (2018).

43 I add.

44 An exception is Terzis, who presents an existentialist critique of AI ethics that depends on individuals’ capacity to reflect on their freedom and responsibilities (Terzis, 2020). Though I sympathize with Terzis’ attempt to bring philosophy in the data and computer science community, I worry that his emphasis on one overarching value to aim for (a transcendental collective form of freedom) is in the end susceptible to the same concerns raised to other principle- and list-based approaches.

45 See e.g. Crawford (2016); Schaap (2006).

46 See for a relevant example of refusal (Barabas, 2020). An argument explaining how ‘saying no’ could even be part of the conceptual apparatus of arguably one of the most consensus-oriented theories of democracy can be found here (White & Farr, 2012).

47 In this context, Noortje Marres writes that the focus on ethical clarity “(…) risks distracting attention and support away from forms of enquiry that are less clearly ‘identifiable,’” like epistemological and methodological issues that new technologies *also* bring about. See Marres (2018, p. 184).

48 Linnet Taylor and Lina Dencik rhetorically ask whether ethics should always be integrated (Taylor & Dencik, 2020, p. 9).

49 Academic ‘unoriginality’ thus does not amount to political irrelevance.

50 The distinction is Foucault’s and is explained here by James Tully. See Tully (2008, p. 103). Foucault talks about it here (Foucault, 1994).

51 Which is still lacking and also difficult to conduct.

52 On the advantages of genealogies, see Prinz and Raekstad (2020).

53 Compare for instance how ‘privacy’ is currently being practiced (Waldman, 2021, Chapter 1).
